# Mapping the diversity of microbial lignin catabolism: experiences from the eLignin database

**DOI:** 10.1007/s00253-019-09692-4

**Published:** 2019-04-08

**Authors:** Daniel P. Brink, Krithika Ravi, Gunnar Lidén, Marie F Gorwa-Grauslund

**Affiliations:** 1 Applied Microbiology, Department of Chemistry, Lund University, P.O. Box 124, SE-221 00 Lund, Sweden; 2 Department of Chemical Engineering, Lund University, Lund, Sweden

**Keywords:** Lignin, Database, Aromatic metabolism, Catabolic pathways, Bioconversion, Ecological niche

## Abstract

Lignin is a heterogeneous aromatic biopolymer and a major constituent of lignocellulosic biomass, such as wood and agricultural residues. Despite the high amount of aromatic carbon present, the severe recalcitrance of the lignin macromolecule makes it difficult to convert into value-added products. In nature, lignin and lignin-derived aromatic compounds are catabolized by a consortia of microbes specialized at breaking down the natural lignin and its constituents. In an attempt to bridge the gap between the fundamental knowledge on microbial lignin catabolism, and the recently emerging field of applied biotechnology for lignin biovalorization, we have developed the eLignin Microbial Database (www.elignindatabase.com), an openly available database that indexes data from the lignin bibliome, such as microorganisms, aromatic substrates, and metabolic pathways. In the present contribution, we introduce the eLignin database, use its dataset to map the reported ecological and biochemical diversity of the lignin microbial niches, and discuss the findings.

## Introduction

**Fig. 1:**
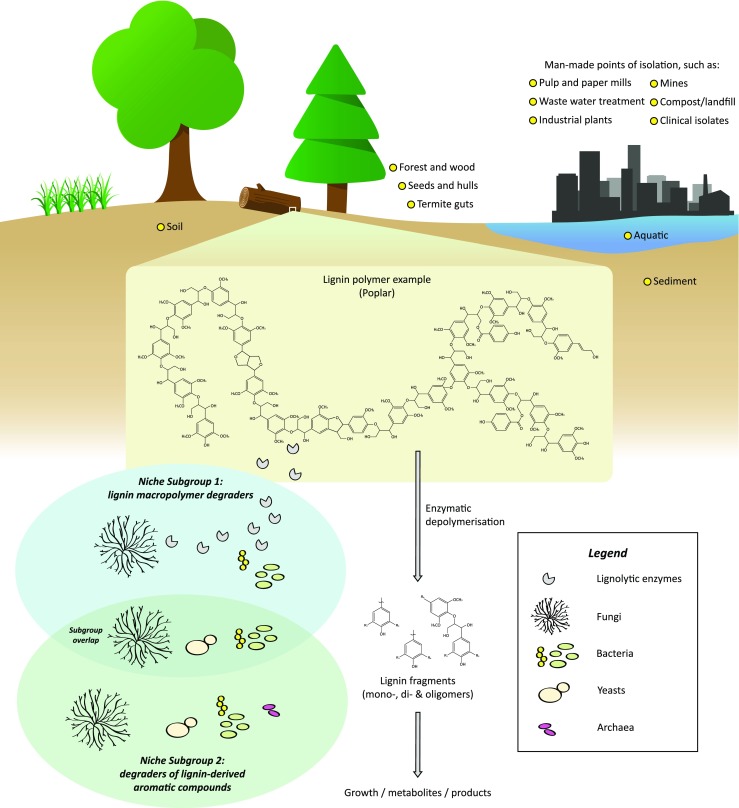
Schematic representation of the lignin microbial niche. In this model of the niche, lignin is mineralized by two subgroups: lignolytic species and aromatic degrading species. Some species degrade or modify lignin to access the hemi-/cellulose on which they grow (subgroup 1), and other species catabolize the aromatic lignin fragments that result from the enzymatic depolymerization (subgroup 2). There is also some overlap between the subgroups, with species capable of both lignolysis and aromatic degradation. Yellow circles represent the different origins of isolation reported for this niche. The poplar lignin structure was adapted from Vanholme et al. (2010)

Lignin is one of the three main components in lignocellulosic biomass and the most abundant terrestrial aromatic macromolecule and is as such a potentially great source of renewable aromatic compounds (Holladay et al. 2007). It is found in the cell walls of lignocellulosic plants (Fig. 1), where it is intertwined with the other two main polymers (cellulose and hemicellulose), and confers structural strength, impermeability, and water transport in the cell wall (Ayyachamy et al. 2013). The main characteristic traits of the lignin macropolymer are its highly amorphous structure—caused by the high heterogeneity of its aromatic building blocks (in turn directly depending on the plant species) (Gellerstedt and Henriksson 2008; Lewis and Yamamoto 1990; Vanholme et al. 2010)—and its severe recalcitrance to chemical and microbial depolymerization (Ruiz-Dueñas and Martínez 2009). Various types of lignin streams (here called *technical lignins*) are produced in high amounts in the pulp and paper industry and are today primarily used to generate process steam and electricity by incineration (Li and Takkellapati 2018; Naqvi et al. 2012). These lignin streams are therefore a largely untapped resource for sustainable production of platform chemicals and have the potential to become a key feedstock in a future expanded biorefinery concepts (Beckham et al. 2016).

Microbial lignin degradation in nature has been studied for decades, with the scientific literature stretching back to at least the 1960s and studies on, e.g., *Pseudomonas putida* (Ornston and Stanier 1966). Due to the high diversity of the lignin heteropolymer, the microbial modes of lignin catabolism are also diverse (Bugg et al. 2011b; Durante-Rodríguez et al. 2018; Fuchs et al. 2011). Lignin degraders are typically bacteria and fungi: among the former, the species mostly belong to the *Actinobacteria* and *Proteobacteria* phyla (Bugg et al. 2011b; Tian et al. 2014); as for the fungi, the common degraders are of the white rot fungi, filamentous fungi, and yeast taxa (Durham et al. 1984; Guillén et al. 2005; Martins et al. 2015). Furthermore, the lignin recalcitrance often prevents one single species from fully degrading the lignin polymer, and instead a symbiosis where rot-type fungi and bacteria are working together is needed to achieve a complete degradation (Cragg et al. 2015; de Boer et al. 2005), thus generating a specific niche (Fig. 1) that selects for a small set of microbial genera.

On the applied side, chemical depolymerization of natural or technical lignins is required to establish a biotechnological value chain from mono- or oligoaromatics. The lignin streams, e.g., from the pulp and paper industry, must be depolymerized to yield mono- and oligomeric aromatic compounds (Ragauskas et al. 2014; Zakzeski et al. 2010) that are then fed to suitable microbes (natural or engineered) for bioconversion into value-added products. However, most knowledge on the microbial side of this process comes from natural degraders, and little is currently known about microbial growth and utilization on the cocktail of aromatic compounds found in depolymerized technical lignin. Furthermore, although different lignocellulosic feedstocks (e.g., softwood, hardwood, agricultural residues) are known to contain different amounts and types of aromatic building blocks (Gellerstedt and Henriksson 2008; Ragauskas et al. 2014), it is very challenging to predict the chemical composition of the mixture resulting from a depolymerization process, especially for technical lignins (Abdelaziz et al. 2016). Consequently, it is difficult to a priori select a suitable microbial host until chemical analysis has been performed on the depolymerized (low molecular weight) lignin stream.

The literature on microbial lignin catabolism is vast and combines fundamental microbiology and applied studies that have in particular seen a surge in popularity during the last decade. However, there has been little effort yet to facilitate an overview of the large amount of publications in this field, especially regarding intracellular microbial events. For this reason, we have created a new database named *The eLignin Microbial Database* (www.elignindatabase.com) for collection of data from scientific literature on the catabolism of lignin and lignin-derived aromatic compound by microorganisms. The eLignin database was launched online in March 2017 and aims to bring together the bibliome of this field in one self-contained searchable platform, and thus fill a gap presently not covered by other online biological databases, as well as to demonstrate the high diversity of this microbial niche (Fig. 1). As the database primarily focuses on intracellular conversion steps, information on extracellular enzymes with lignolytic activities are currently not covered and the readers are redirected to, e.g., the following reviews (Janusz et al. 2017; Sigoillot et al. 2012).

The present minireview will introduce the design philosophy of the eLignin database and present our outcome of the diversity analysis with prime focus on intracellular microbial events. What sets this paper apart from other recent reviews discussing the diversity of microbial lignin degradation (Bugg et al. 2011a; Tian et al. 2014) is that we have been able to use the established content of the database (Table 1) to make pattern recognitions over the indexed publications in eLignin (for instance using relational SQL queries and Python scripts).

**Table 1:** Content of the eLignin database as of the time of writing

Entry	Count
Organisms	261 organisms (171 prokaryotes, 85 eukaryotes, 5 archaea)
Substrates	141
Metabolic pathways	26
Genes	90
Enzymes	59
Reactions	76
Total entries	653
References	330

Please note that these figures are subject to increase over time, as more data and references (both past and newly published scientific literature) are continuously added

## Scope and design of the eLignin database

The eLignin database was created because there is, to our knowledge, no currently available database dedicated to microbial lignin catabolism. A literature survey showed that there have been published databases on lignin biochemistry in the past, but they are, at the time of writing, all unavailable and/or discontinued: FOLy, a database on fungal oxidoreductases for lignin catabolism (Levasseur et al. 2008); LD^2^L, a database similar in scope as eLignin (Arumugam et al. 2014); and an NMR database for lignin structures (Ralph et al. 2004), with the latter not treating microbial catabolism. The objective of eLignin is to collect data on strains of microorganisms (bacteria, yeasts, and fungi) known to degrade and/or catabolize lignin and lignin-derived aromatic compounds. Specifically, the database content includes microorganisms, substrates, pathways, genes, metabolic reactions, and enzymes related to the topic (Table 1). So far, its prime focus has been on collecting data on microbial diversity and *intracellular* events; however, the database can later be expanded with *extracellular* enzymes and reactions (such as laccases and peroxidases), as these play an important role in microbial degradation of native lignin and can be applied for enzymatic depolymerzation of technical lignins (Bourbonnais et al. 1995; Pardo et al. 2018; Zhao et al. 2016).

In practice, the data in eLignin is retrieved from scientific literature (peer-reviewed articles, reviews, and books), manually curated and supplemented with links to relevant entries in other well-established biological and chemical databases (e.g., GenBank (Benson et al. 2012), KEGG (Kanehisa and Goto 2000), PubChem (Kim et al. 2015), and ChEBI (Hastings et al. 2012)). The initial dataset was collected by performing a *systematic literature review* according to the Kitchenham protocol (Kitchenham 2004), where 561 articles (title, abstract, and keywords) were screened and analyzed for their inclusion in the database *bibliome*. Since the eLignin dataset originates from scientific literature, users are encouraged to read the primary references for any data of interest, since there will be aspects of the data that are not indexed or reviewed by eLignin (such as experimental conditions). Due to the nature of the data collection for eLignin (scientific publications), there will be some overlap with other biological databases such as MetaCyc (Caspi et al. 2015), GenBank (Benson et al. 2012), or UniProt (UniProtConsortium 2017), when it comes to information on pathways, genes, and enzymes. As we do not strive to master features that already established databases already do, eLignin entries are annotated with links to specialized databases where possible.

**Fig. 2:**
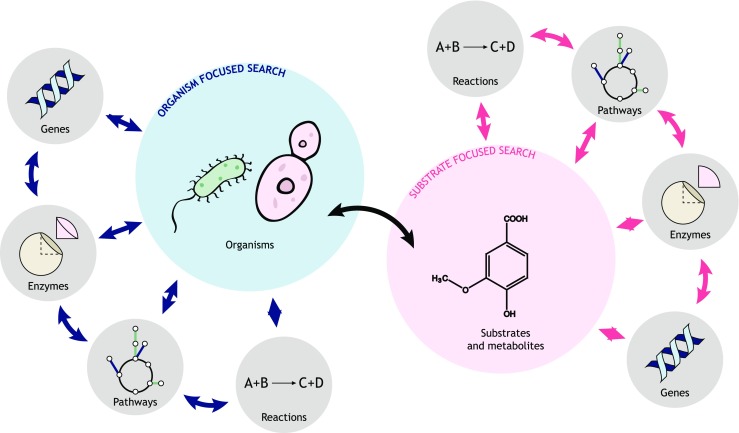
Schematic overview of the eLignin database. The figure illustrates that eLignin is *a microorganism- and substrate-focused database* and that every entry type (organism, substrate, gene, enzyme, pathway, reaction) is accessible from each of these point-of-entries

Two major entry points were considered for eLignin: a microorganism- and a substrate-oriented search (Fig. 2). This design choice was made in order to cater to what we foresee are the two most common information needs both in fundamental and applied lignin microbial conversion: (i) What substrates can my microbe of choice breakdown and/or utilize?; (ii) What microorganism can I use to consume the lignin and lignin-derived aromatics in my substrate stream? Using these entry points, we will now describe the current state of the bibliome and use eLignin content to map and discuss the presently known diversity of the lignin microbial niche.

## The microbial diversity in the lignin niche, as reported in the eLignin bibliome

Lignocellulose degradation through cellulolytic activity has been found to be distributed in a wide range of genera within the Bacteria, Archaea, Fungi, and Animalia kingdoms (Cragg et al. 2015). However, the known lignin-degrading subset of lignocellulose degraders is so far limited to a few bacterial and fungal phyla (Janusz et al. 2017; Tian et al. 2014). Mineralization of the lignin requires two main steps: (1) breakdown of the lignin macropolymer to yield smaller aromatic compounds and (2) ring fission of the resulting aromatic compounds (Tuor et al. 1995). The first step is carried out by microbes able to secrete extracellular enzymes with lignolytic and/or lignin-modifying activities such as laccases and peroxidases—typically wood-decaying fungi and certain bacterial species (Bugg et al. 2011b; Janusz et al. 2017; Sigoillot et al. 2012) (Fig. 1). The resulting heterogeneous mixture of aromatic breakdown products is then metabolized by the lignolytic secreters themselves or by other microorganisms in the vicinity capable of aromatic catabolism (Cragg et al. 2015). This leads to the establishment of a microbial niche that favors microbes with matching substrate specificity for the resulting aromatic compounds and with tolerance to the often inhibitory or toxic nature of the aromatics (Díaz et al. 2013; Krell et al. 2012; Schweigert et al. 2001). During catabolism, the aromatic breakdown products are typically shunted through a number of reactions that are collectively referred to as *funneling pathways* (Harwood and Parales 1996)—or sometimes *upper pathways* (Linger et al. 2014)—that eventually converge on a couple of conserved ring fission pathways where the aromatic rings are cleaved and the subsequent metabolites enter the central carbon metabolism (Fuchs et al. 2011). Because of these two main steps (depolymerization and ring fission), the lignin microbial niche can be said to contain two main groups of microbes: lignin macropolymer degraders and degraders of lignin-derived aromatic compounds (with the former often being capable of the latter (Nakamura et al. 2012)), from here on referred to as niche subgroups 1 and 2 (Fig. 1). The eLignin database aims to index both, and for the remainder of the minireview, the concept of the *lignin microbial niche* will be used to refer to all microbes that are capable of degrading lignin and lignin-derived aromatic compounds. Subgroup 2 is of importance for applied studies aiming to, e.g., valorize chemically depolymerized lignin, or to perform in situ bioremediation, and thus, an extra effort has been put on this group in the eLignin database.

Within the applied side of lignin bioconversion, a quick survey of the recent literature shows that a substantial amount of research articles focus on a few commonly used model organisms such as *Pseudomonas putida* (Linger et al. 2014), *Sphingobium* sp. (Masai et al. 1999), *Rhodococcus jostii* (Sainsbury et al. 2013), and *Rhodococcus opacus* (Kosa and Ragauskas 2012). Reviews on microbial lignin degradation that often include large tables with important isolates (Abdelaziz et al. 2016; Bugg et al. 2011a; Tian et al. 2014) are seldom listing more than ~ 50 different microbes. Still, over 250 microorganisms with lignin and lignin-derived aromatics catabolic activity are currently mapped in the eLignin bibliome (Tables 1, 2, and 3), which indicates its usefulness for meta-analysis of the field.

**Table 2:** Distribution of bacterial genera in the dataset of known degraders of lignin and/or lignin-derived aromatics index in the eLignin database

Genus (sorted by phylum)	Number of species in eLignin	References
*Acidobacteria* (Gram stain differs with species)		
*Holophaga*	1	Bak et al. (1992)
*Actinobacteria* (Gram-positive)		
*Amycolatopsis*	1	Sutherland (1986)
*Arthrobacter*	1	Kerr et al. (1983)
*Corynebacterium*	1	Qi et al. (2007)
*Microbacterium*	1	Song (2009), Taylor et al. (2012)
*Micrococcus*	1	Taylor et al. (2012)
*Nocardia*	2	Crawford et al. (1973), Kuhnigk and Konig (1997)
*Pelobacter*	1	Schink and Pfennig (1982)
*Rhodococcus*	11	Chong et al. (2018), Chung et al. (1994), Eulberg et al. (1997), Henson et al. (2018), Karlson et al. (1993), Kosa and Ragauskas (2012), Sainsbury et al. (2013), Song (2009), Taylor et al. (2012)
*Streptomyces*	10	Antai and Crawford (1981), Aoyama et al. (2014), Chow et al. (1999), Davis and Sello (2010), Giroux et al. (1988), Ishiyama et al. (2004), Kuhnigk and Konig (1997), Watanabe et al. (2003), Yang et al. (2012), Zeng et al. (2013)
*Thermobifida*	1	Chang et al. (2014)
*Thermomonospora*	1	McCarthy and Broda (1984)
*Bacteroidetes* (Gram-negative)		
*Dysgonomonas*	1	Duan et al. (2016b)
*Sphingobacterium*	1	Taylor et al. (2012)
*Firmicutes* (mostly Gram-positive)		
*Acetoanaerobium*	1	Duan et al. (2016a)
*Acetobacterium*	2	Bache and Pfennig (1981), Kaufmann et al. (1998)
*Aneurinibacillus*	1	Raj et al. (2007)
*Bacillus*	10	Chandra et al. (2007), Huang et al. (2013), Kuhnigk and Konig (1997), Perestelo et al. (1989), Zhu et al. (2017)
*Brevibacillus*	2	Hooda et al. (2015, 2018)
*Clostridium*	2	Daniel et al. (1988), Mechichi et al. (1999)
*Paenibacillus*	3	Chandra et al. (2007), Crawford et al. (1979), Mathews et al. (2014)
*Papillibacter*	1	Defnoun et al. (2000)
*Spirochaetes* (Gram stain differs with species)		
*Treponema*	1	Lucey and Leadbetter (2014)
*Proteobacteria* (Gram-negative)		
*Achromobacter*	1	Benjamin et al. (2016)
*Acinetobacter*	7	Delneri et al. (1995), Fischer et al. (2008), González et al. (1993), Kuhnigk and Konig (1997), Mazzoli et al. (2007), Van Dexter and Boopathy (2018), Vasudevan and Mahadevan (1992)
*Aeromonas*	2	Deschamps et al. (1980), Gupta et al. (2001)
*Agrobacterium*	1	Parke (1997)
*Alcaligenes*	1	Kuhnigk and Konig (1997)
*Aromatoleum*	1	Rabus and Widdel (1995)
*Azoarcus*	1	Gorny et al. (1992)
*Azotobacter*	2	Hirose et al. (2013), Groseclose and Ribbons (1981)
*Bradyrhizobium*	1	Sudtachat et al. (2009)
*Burkholderia*	7	Hamzah and Al-Baharna (1994), Harazono et al. (2003), Kato et al. (1998), Kuhnigk and Konig (1997), Song (2009), Woo et al. (2014b), Yang et al. (2017)
*Citrobacter*	3	Chandra and Bharagava (2013), Harazono et al. (2003)
*Comamonas*	5	Chen et al. (2012c), Kamimura et al. (2010), Kuhnigk and Konig (1997), Ni et al. (2013), Providenti et al. (2006)
*Cupriavidus*	5	Hughes and Bayly (1983), Perez-Pantoja et al. (2008), Sato et al. (2006), Shi et al. (2013a)
*Desulfobacterium*	2	Bak and Widdel (1986), Szewzyk and Pfennig (1987)
*Enterobacter*	5	DeAngelis et al. (2013), Deschamps et al. (1980), Grbić-Galić (1985), Yoshida et al. (2010)
*Flavimonas*	1	Song (2009)
*Flavobacterium*	1	Hirose et al. (2013)
*Klebsiella*	4	Hirose et al. (2013), Jones and Cooper (1990), Woo et al. (2014a), Xu et al. (2018)
*Marinobacterium*	1	González et al. (1997)
*Mesorhizobium*	1	Tian et al. (2016)
*Microbulbifer*	1	González et al. (1997)
*Moraxella*	1	
*Novosphingobium*	2	Chen et al. (2012b), Liu et al. (2005)
*Oceanimonas*	1	Numata and Morisaki (2015)
*Ochrobactrum*	6	Hirose et al. 2013), Kuhnigk and Konig (1997), Taylor et al. (2012), Tsegaye et al. (2018), Xu et al. (2018)
*Pandoraea*	3	Bandounas et al. (2011), Kumar et al. (2015), Shi et al. (2013b)
*Pantoea*	3	Song (2009), Xiong et al. (2014), Zeida et al. (1998)
*Pseudomonas*	27	Chapman and Ribbons (1976), Chowdhury et al. (2004), Cronin et al. (1999), Gao et al. (2005), Hirose et al. (2013), Hirose et al. (2018), Iwabuchi et al. (2015), Jimenez et al. (2002), Jurková and Wurst (1993), Kuhnigk and Konig (1997), Li et al. (2010), Mahiudddin and Fakhruddin (2012), Maruyama et al. (2004), Murray et al. (1972), Narbad and Gasson (1998), Nikodem et al. (2003), Ornston and Parke (1976), Overhage et al. (1999), Perestelo et al. (1996), Ravi et al. (2018), Shettigar et al. (2018), Tian et al. (2016), Xu et al. (2018)
*Rhizobium*	1	Jackson et al. (2017)
*Rhodopseudomonas*	2	Harwood and Gibson (1988), Salmon et al. (2013)
*Sagittula*	1	Gonzalez et al. (1997)
*Serratia*	5	Haq et al. (2016), Perestelo et al. (1990), Rhoads et al. (1995), Tian et al. (2016)
*Sinorhizobium*	1	MacLean et al. (2006)
*Sphingobium*	1	Masai et al. (2007)
*Sphingomonas*	1	Balkwill et al. (1997)
*Stenotrophomonas*	1	Tian et al. (2016)
*Sulfuritalea*	1	Sperfeld et al. (2018)
*Thauera*	2	Mechichi et al. (2005), Tschech and Fuchs (1987)
*Tolumonas*	1	Billings et al. (2015)
*Trabulsiella*	1	Suman et al. (2016)

**Table 3:** Distribution of fungal genera in the dataset of known degraders of lignin and/or lignin-derived aromatics index in the eLignin database

Genus (sorted by phylum)	Number of species in eLignin	References
*Ascomycota*		
*Aspergillus*	3	Barapatre and Jha (2017), Martins et al. (2015), Yang et al. (2011)
*Brettanomyces*	1	Edlin et al. (1995)
*Candida*	7	Fialova et al. (2004), Gérecová et al. (2015), Krug et al. (1985)
*Emericella*	1	Barapatre and Jha (2017)
*Exophiala*	1	Middelhoven (1993)
*Fusarium*	5	Chang et al. (2012), Falcon et al. (1995), Korniłłowicz-Kowalska and Rybczyńska (2015), Michielse et al. (2012)
*Oudemansiella*	1	Fukasawa et al (2011)
*Geotrichum*	1	Sláviková and Košíková (2001)
*Penicillium*	1	Rodriguez et al. (1994)
*Pestalotia*	1	Falcon et al. (1995)
* Petriellidium*	1	Eriksson et al. (1984)
*Phialophora*	1	Eriksson et al. (1984)
*Phoma*	1	Bi et al. (2016)
*Trichoderma*	3	Korniłłowicz-Kowalska and Rybczyńska (2015), Ryazanova et al. (2015)
*Basidiomycota*		
*Agaricus*	1	Saha et al. (2016)
*Anthracophyllum*	1	Acevedo et al. (2011)
*Auricularia*	1	Liers et al. (2011)
*Bjerkandera*	3	Fukasawa et al. (2011), Liers et al. (2011), Saha et al. (2016)
*Ceriporiopsis*	1	Rüttimann-Johnson et al. (1993)
*Cryptococcus*	1	Bergauer et al. (2005)
*Cyathus*	3	Saha et al. (2016), Sethuraman et al. (1999)
*Daedalea*	1	Arora and Sandhu (1985)
*Hymenochaete*	1	Saito et al. (2018)
*Dichomitus*	1	Périé and Gold (1991)
*Irpex*	2	Saha et al. (2016), Xu et al. (2009)
*Leucosporidium*	1	Middelhoven (1993)
*Marasmius*	1	Saito et al. (2018)
*Mastigobasidium*	1	Bergauer et al. (2005)
*Microbotryomycetidae*	1	Bergauer et al. (2005)
*Mycena*	1	Liers et al. (2011)
*Nematoloma*	1	Hofrichter et al. (1999)
*Phanerochaete*	4	Eriksson et al. (1983), Hiratsuka et al. (2005), Saha et al. (2016), Vares et al. (1994)
*Phlebia*	5	Bi et al. (2016), Liers et al. (2011), Saito et al. (2018), Vares et al. (1994)
*Pleurotus*	1	Liers et al. (2011)
*Polyporus*	1	Saha et al. (2016)
*Pycnoporus*	3	Eggert et al. (1996), Saha et al. (2016)
*Rhodosporidium*	2	Bergauer et al. (2005), Yaegashi et al. (2017)
*Rhodotorula*	8	Bergauer et al. (2005), Durham et al. (1984), Gupta et al. (1986), Hainal et al. (2012), Huang et al. (1993), Sampaio (1999)
*Rigidoporus*	1	Saha et al. (2016)
*Sporobolomyces*	1	Bergauer et al. (2005)
*Stropharia*	2	Liers et al. (2011), Saito et al. (2018)
*Trametes*	3	Alexieva et al. (2010), Fukasawa et al. (2011), Knežević et al. (2018)
*Trichosporon*	4	Middelhoven 1993), Sietmann et al. (2001), Sláviková et al. (2002), Yaguchi et al. (2017)

The listed species in the current dataset of eLignin are distributed over 90 different genera, which in turn can be classified into six bacterial phyla (*Acidobacteria*, *Actinobacteria*, *Bacteroidetes*, *Firmicutes*, *Proteobacteria*, and *Spirochaetes*) and two fungal phyla (*Ascomycota* and *Basidiomycota*) (see Tables 2 and 3). However, the majority of the microbes belong to five of the eight observed phyla: *Proteobacteria* (114 species/strains), *Basidiomycota* (58 species/strains), *Actinobacteria* (31 species/strains), *Ascomycota* (27 species/strains), and *Firmicutes* (22 species/strains) (Tables 2 and 3). Evidence of some aromatic-degrading archaea (of the kingdom of *Euryarchaeota*) is also beginning to emerge (Emerson et al. 1994; Erdoğmuş et al. 2013; Khemili-Talbi et al. 2015). Overall, the large occurrence of *Proteobacteria* is noteworthy, and the species of this phylum are indeed enriched in studies of isolates found from lignin-rich environments and selected on growth on lignin and aromatic compounds (Jimenez et al. 2002; Jurková and Wurst 1993; Kuhnigk and Konig 1997; Narbad and Gasson 1998; Overhage et al. 1999; Perestelo et al. 1996; Ravi et al. 2017). Likewise, when the same organisms were analyzed for their origin of isolation, it was clear that a majority originated from soil and from the forest ground layer (Table 4), which is probably the most expected ecosystem for the lignin microbial niche (Cragg et al. 2015; Harwood and Parales 1996) given the abundance of lignocellulose in different states of decay found in there.

**Table 4:** Origin of isolation of the 261 organisms listed in the eLignin database as of November 2018

Origin of isolation	Number of organisms			
	Total	Bacteria	Fungi	Archaea
Aquatic	8	5	1	2
Caves and mines	6	1	5	0
Clinical isolate	6	0	6	0
Compost	5	5	0	0
Forest and wood samples	40	17	23	0
Industrial plants	5	1	2	2
Lab-made derivative	4	4	0	0
Other	2	1	1	0
Pulp and paper mill effluent	16	15	1	0
Sediment	15	15	0	0
Seeds and hulls	2	2	0	0
Soil	92	70	21	1
Termite gut	22	22	0	0
Unknown or not specified	27	3	24	0
Wastewater sludge	11	10	1	0

The organisms have been sorted in 15 main clusters in order to facilitate the clustering, and the specific details can be found in the database entry for each organism

The following subsections will discuss the outcome of the analysis of the database content in terms of fungal, bacterial, and archaeal diversity. Also, in order to complement the pure isolate approach of the database, the last subsection will discuss microbial communities.

### Fungal diversity

The fungi listed in the database are either of the wood rot-type or yeasts. Wood-decaying, or wood-rot, fungi are found within the *Basidiomycota* and *Ascomycota* phyla and can be divided into three different types that all have lignin-modifying activities to various extent: soft-rot, brown-rot, and white-rot fungi (Hatakka 2005; Janusz et al. 2017). Soft-rot fungi tend to prefer hardwood and seem to only weakly affect lignin (Sigoillot et al. 2012), but a few species have been reported to exhibit white-rot–like activity toward the end of the wood decay (Pildain et al. 2005). Brown-rot fungi, which are mainly found in the *Basidiomycota* phylum, selectively attack hemicellulose and cellulose and leave a modified (e.g., dealkylated, demethoxylated, and/or demethylated) lignin signified by its brown color (hence the name of this group of wood degraders); they are primarily found in softwood ecosystems (Hatakka 2005; Sigoillot et al. 2012). Finally, white-rot fungi can degrade all three main components of lignocellulose, i.e., hemicellulose, cellulose, and lignin, and leave a decayed wood with a bleached color (Blanchette 1984; Eriksson et al. 1980; Sigoillot et al. 2012). White-rots are the only wood-decaying fungi that can completely degrade lignin to CO_2_ and H_2_O; however, it has been proposed that lignin cannot be used as the sole carbon source by white-rots; rather, the lignin degradation is probably a process that the fungi use to access the cellulose and hemicellulose (ten Have and Teunissen 2001). Like brown-rot fungi, white-rot fungi mostly belong to the *Basidiomycota* phylum and to a smaller extent to the *Ascomycota* (Sigoillot et al. 2012).

Both brown-rot and white-rot fungi invade the wood cell lumen by hyphal growth and secrete their lignocellolytic enzymes (Kirk and Farrell 1987; Leonowicz et al. 1999). The lignolytic mechanisms of white-rot fungi secretome have been thoroughly studied (Leonowicz et al. 1999; ten Have and Teunissen 2001). The known lignolytic enzymes (e.g., lignin peroxidases, manganese peroxidases, versatile peroxidases, and laccases (Janusz et al. 2017)) work by nonspecific oxidation, and although nucleophilic cleavage can be used for chemical depolymerization of lignin (e.g., in kraft pulping), the highly variable tertiary structure of lignin could explain why no nucleophilic lignolytic enzymes have been described (Hammel and Cullen 2008). The level and patterns of decay vary between different fungal species and the type of wood (Worrall et al. 1997) as well as the state of decay of the wood. Fukasawa and colleagues subjected beech wood in varying levels of decay to different fungal species and were able to demonstrate that the *Basidiomycota* caused its highest weight loss in nondecayed wood, whereas the assayed *Ascomycota* caused more weight loss in predecayed wood (Fukasawa et al. 2011), which implicates that there is a sequential order within the lignin microbial niche with different types of fungi taking turns for degrading the (residual) wood. A very thorough indexing of microbes (primarily fungi) that secrete lignolytic enzymes can be found in a supplemental table of the review of Janusz et al. (2017).

The 85 eukaryotes (fungi and yeasts from 43 different genera) currently listed in eLignin are distributed between *Ascomycota* and *Basidiomycota* (Table 3) and were primarily isolated from soil and forest environments (Table 4), which is in accordance with other reviews of the ecological occurrence (Janusz et al. 2017). The clinical isolates reported in Table 4 are mainly different species of *Candida* yeasts, which aside from their opportunistic pathogenicity in humans, are known degraders of lignocellulose-derived compounds such as xylose and different aromatics (Gérecová et al. 2015; Holesova et al. 2011; Jeffries 1981; Krug et al. 1985). In general, the yeasts species in the database are aromatic degraders and not lignin degraders (Bergauer et al. 2005; Holesova et al. 2011; Middelhoven 1993; Yaegashi et al. 2017) and, therefore, play a role in the niche as degraders of lignin breakdown products. Three species in the dataset have, however, been reported to have activity on lignin: *Rhodotorula* sp. R2 modified wheat straw and Sarkanda grass (Hainal et al. 2012), whereas *Geotrichum klebahnii* CCY 74-6-2 and *Trichosporon pullulans* CCY 30-1-10 acted on beechwood lignin fractionated from the prehydrolysis step of kraft pulping (Sláviková and Košíková 2001; Sláviková et al. 2002).

When it comes to lignin-degrading activity, fungi tend to be more studied than bacteria because of their higher prevalence of lignolytic secretomes (Janusz et al. 2017). However, if the system boundaries are expanded to include the whole lignin aromatic niche, i.e., the species that lack delignification activities but grow on the lignin-derived aromatic compounds (Fig. 1), the ratio between fungi and bacteria could be rather different. In eLignin, which was built on this niche principle, there are about two times as many bacterial isolates listed as fungal ones (Tables 1, 2, and 3). We cannot determine if this is a bias in the literature, comes from the database boundaries (which were initially created with a focus on intracellular events, and not on secreted enzymes), or if the “true” diversity holds less fungal species than bacterial. The number of wood-rotting Basidiomycetes has been estimated to up to 1700 species in North America only, but the number of lignolytic fungi is unknown (Gilbertson 1980; Janusz et al. 2017).

### Bacterial diversity

By using the holistic ecological approach to list both degraders of lignin and lignin-derived aromatic compounds, 171 different bacteria distributed over 63 different genera have been indexed in eLignin at the time of writing (Table 2). As mentioned above, three main phyla encompasses the bulk of the dataset (*Proteobacteria*, *Actinobacteria*, and *Firmicutes*), with *Proteobacteria* dominating the list with its 114 entries (Table 2). Within these *Proteobacteria*, γ-*Proteobacteria* was the main class (66 species/strains), followed by β-*Proteobacteria* (27 species/strains), α-*Proteobacteria* (18 species/strains), and δ-*Proteobacteria* (3 species/strains), again highlighting that certain types of microbes are greatly enriched in the eLignin bibliome. It can also be noted that many of the organisms in this particular niche have undergone one or several taxonomical reclassifications since they were first isolated and described (see, e.g., *Cupriavidus necator* which was previously known as, e.g., *Ralstonia eutropha* and *Wautersia eutropha* (Vandamme and Coenye 2004)), meaning that the binomial names in articles from the 1960–1980s may be different from the currently prevailing names. Therefore, the organism entry in the database has, when possible, been harmonized with links to the corresponding entry in the NCBI Taxonomy Database (https://www.ncbi.nlm.nih.gov/taxonomy; Acland et al. 2014).

The Gram stain distribution tends to follow the phyla and, thus, is dominated by Gram-negative bacteria (121 species/strains), with the remainder being Gram-positive (46 species/strains) and unknown/Gram-indeterminate (4 species/strains). This may have implication on studies focusing on, e.g., transport of compounds over membranes (discussed in a separate section below), or when expanding a species’ substrate range by metabolic engineering. In the latter case, the difference in total GC content in the genome that is in general seen between Gram-positives and Gram-negatives (Muto and Osawa 1987) will affect the feasibility of heterologous expression if using traditional PCR-based cloning.

Although fungi are known as the main degraders of the lignin macropolymer (as described in the previous subsection), there are a substantial number of studies that describe delignifying bacteria. Tian et al. reviewed the topic and performed phylogeny on 57 lignin-degrading and 463 laccase-encoding prokaryotes that led them to propose that screening for laccases genes may be a good way to detect new lignin-degrading species (Tian et al. 2014). Furthermore, the authors suggest that aromatic metabolism is a prerequisite for but not a proof of lignolytic activity (Tian et al. 2014), which is in line with our division of the lignin bacterial niche into subgroups 1 and 2 that specialize in different aspects of the full lignin catabolism (Fig. 1). The metabolism of the resulting lignin breakdown products, which mainly takes place intracellularly, will be discussed in the “Distribution of metabolic pathways and substrate specificities” section below.

Soil is absolutely the most common origin of isolation mapped in the database (Table 4), which also reflects how popular this environment has been for studies on isolation of lignin and aromatic degraders. Other than soil, termite guts are a main origin of isolation. There seems to be no clear evidence that the termites themselves are able to degrade lignin (instead they live of the hydrolysis products of hemicellulose and cellulose) (Brune and Ohkuma 2010). The lignin barrier is overcome by the termites by a symbiotic relationship with a diverse microbial community, e.g., by exosymbiotic fungi and endosymbiotic gut flora (Maurice and Erdei 2018). Examples of aromatic degrading bacteria isolated from the gut flora include *Proteobacteria* (Harazono et al. 2003; Kuhnigk and Konig 1997; Suman et al. 2016; Tsegaye et al. 2018; Van Dexter and Boopathy 2018), *Actinobacteria* (Chung et al. 1994; Kuhnigk and Konig 1997; Watanabe et al. 2003), and *Firmicutes* (Kuhnigk and Konig 1997), as well as the only *Spirochaetes* entry in the database (Lucey and Leadbetter 2014). Another enrichment reported in Table 4 for bacteria is the isolates from different man-made environments. One example is pulp and paper mill effluents that contain residual lignins and aromatics and have been a source of many isolates (Chandra et al. 2007; Duan et al. 2016b; González et al. 1997; Hooda et al. 2015; Mathews et al. 2014; Nishikawa et al. 1998; Ravi et al. 2018); likewise, sludge from waste water treatment plants has been a source of a number of isolates, some of which are strictly anaerobic (Gorny et al. 1992; Mechichi et al. 1999, 2005; Ni et al. 2013; Traunecker et al. 1991; Tschech and Fuchs 1987).

Anaerobic aromatic degrading bacteria are in a minority compared to the aerobic fission bacteria and were even for a long time believed to be impossible (Kirk and Farrell 1987). However, with recent advances in the field, the molecular biology of these pathways has begun to be understood (Durante-Rodríguez et al. 2018). Some examples found in the database include, e.g., *Pelobacter acidigallici* Ma Gal2 (Schink and Pfennig 1982), *Desulfobacterium phenolicum* Ph01 (Bak and Widdel 1986), *Rhodopseudomonas palustris* CGA001 (Harwood and Gibson 1988), *Clostridium thermoaceticum* ATCC 39073 (Daniel et al. 1988), and *Dysgonomonas* sp. WJDL-Y1 (Duan et al. 2016b); *Holophaga foetida* TMBS4 is also worthy of mention as it the only observed species in the *Acidobacteria* phylum reported in the database, and it grows anaerobically on a couple of typically lignin-derived aromatics such as ferulic acid and syringic acid (Bak et al. 1992).

### Archaeal diversity

Of the three domains in the Woeseian system (Woese et al. 1990), archaea is the most underrepresented in the lignin microbial niche. To our knowledge, there are no reported archaeal single culture isolates with lignolytic capacity at the time of writing. Recently, by enrichment cultures from estuarine sediment, it was possible to infer growth of *Bathyarchaeota* on alkali lignin by the increase in gene-copy number and the incorporation of inorganic carbon in the archaeal lipids over 11 months (Yu et al. 2018). Likewise, putative laccase genes have been reported in some archaeal species (Ausec et al. 2011; Sharma and Kuhad 2009; Tian et al. 2014). A laccase from *Haloferax volcanii* DS70 has been purified with activity on model compounds such as syringaldazine and ABTS (Uthandi et al. 2010). However, to our understanding, the in vivo lignolytic activity of these putative and purified laccases remains to be assayed.

Five archaeal isolates—classified in niche subgroup 2 (growth on aromatics; Fig. 1)—have so far been indexed in eLignin, all of them being halophiles, i.e., extremophiles that prefer high salt concentration. *Haloferax* sp. D1227 was isolated from soil and grew on benzoic, cinnamic, and phenylpropanoic acid (Emerson et al. 1994). *Haloferax* sp. C-24, *Halorubrum ezzemoulense* C-46, and *Haloarcula* sp. D1 were isolated from high-saline samples and grew on, e.g., 4-hydroxybenzoic acid (Erdoğmuş et al. 2013; Fairley et al. 2002). *Natrialba* sp*.* C21 degraded phenol (Khemili-Talbi et al. 2015). The halophilic nature of these isolates and the lack of known lignolytic activity seem to suggest that they contribute with the degradation of aromatic breakdown products that have ended up in saltwater environments, which could be speculated to be a downstream (or *downriver*) extension of the lignin microbial niche.

### The communities of the lignin microbial niche

Lignin degradation is a community effort and is in itself often a subpart of a lignocellulose-degrading niche (de Boer et al. 2005). Microbial communities—organisms that live and interact within a contiguous environment (Konopka 2009)—are in a way what we are illustrating by looking at the isolates from the point of the niche subgroups (Fig. 1). It has been proposed that lignin degradation is more rapid with consortia than single isolates due to synergism (Wang et al. 2013). Furthermore, studies on fungal–bacterial interactions in the lignin microbial niche have reported examples of commensalism as well as amensalism between certain species: some bacteria have been reported to promote growth of a white-rot fungi when co-cultivated (Harry-asobara and Kamei 2018), and there is a report showing two different white-rot species outcompeting opportunistic bacteria (Folman et al. 2008). At the moment, consortia are not mapped in eLignin but are nevertheless important for the understanding of the lignin microbiology.

Many studies have reported physiological characterization of a community with unknown or partly known composition, either because it was not possible to isolate single cultures with the desired phenotype—for instance, 99% of the bacteria in soil have been estimated to be unculturable (Pham and Kim 2012)—or because the aim was to study the community effort. Examples include communities capable of degrading lignin (DeAngelis et al. 2011; Wang et al. 2013; Wu and He 2013), syringic acid (Kaiser and Hanselmann 1982; Phelps and Young 1997), resorcinol and catechol (Milligan and Häggblom 1998), coniferyl alcohol (Grbić-Galić 1983), and plant lignin–soil community studies (Bennett et al. 2015; Bradley et al. 2007), to name a few. Many of these studies were reported under anaerobic conditions.

Another approach to analyze microbial communities is to consider the makeup of the metagenome as a unique property of a given community (Konopka 2009). 16S rRNA sequencing can be used to taxonomically identify members of a community (González et al. 1996). A common methodology is to divide the results of the 16S rRNA sequencing of a metagenome into operational taxonomic units (OTUs) to attempt to resolve, e.g., phylum level abundances (Moraes et al. 2018); this is similar to what is done here with the eLignin database using single isolates (Tables 2 and 3). In addition to taxonomical metagenomics, Moraes and colleagues reconstructed draft bacterial genomes from a lignin-degrading consortium and could identify conserved domains related to lignin degradation in their metagenome (Moraes et al. 2018).

## Distribution of metabolic pathways and substrate specificities

**Fig. 3:**
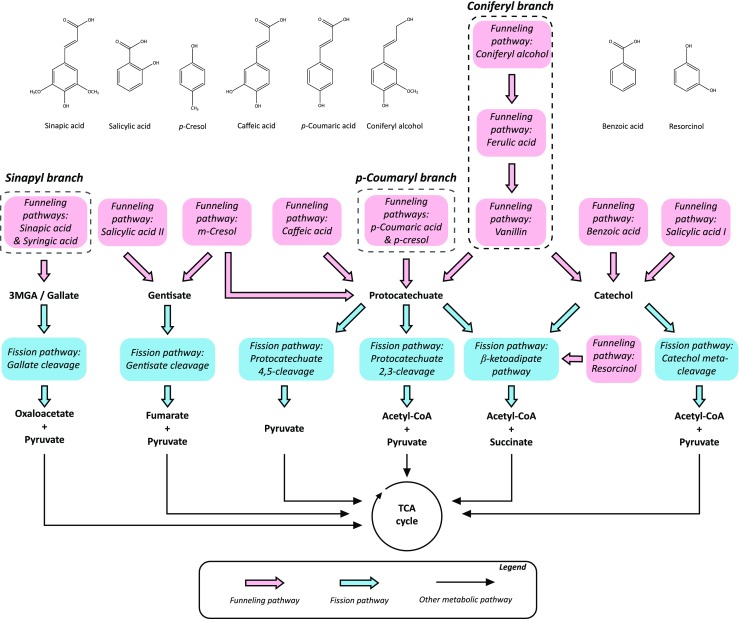
Schematic distribution of the known pathways for aromatic catabolism currently indexed in the eLignin database. Please note that this representation should be seen as a hypothetical map of the existing possibilities within aromatic catabolism, and not as a map of a “superbug.” *Funneling pathways* refer to routes that reduce larger/more substituted aromatic compounds down to the different catabolic nodes from where ring fission occurs (here called *fission pathways*). The three routes that funnel compounds derived from the primary monolignols (S, H, G) are indicated in dotted boxes: the sinapyl (S), *p-*coumaryl (H), and coniferyl (G) branches

The lignin macropolymer is primarily depolymerized by extracellular enzymes secreted by lignolytic microbes. Due to its heterogeneity, the resulting depolymerization products are commonly a mixture of different mono- and di- and oligoaromatic compounds (Bugg et al. 2011b). This has led to the evolution of a panel of intracellular *funneling pathways*, i.e., metabolic routes that connect substituted aromatic compounds with a ring fission pathway leading to the central carbon metabolism, often (but not always) via acetyl-CoA (Fig. 3). In this section, the eLignin database was used to assess the diversity of substrates and metabolic routes within the lignin microbial niche.

### Reported substrate specificities

**Fig. 4:**
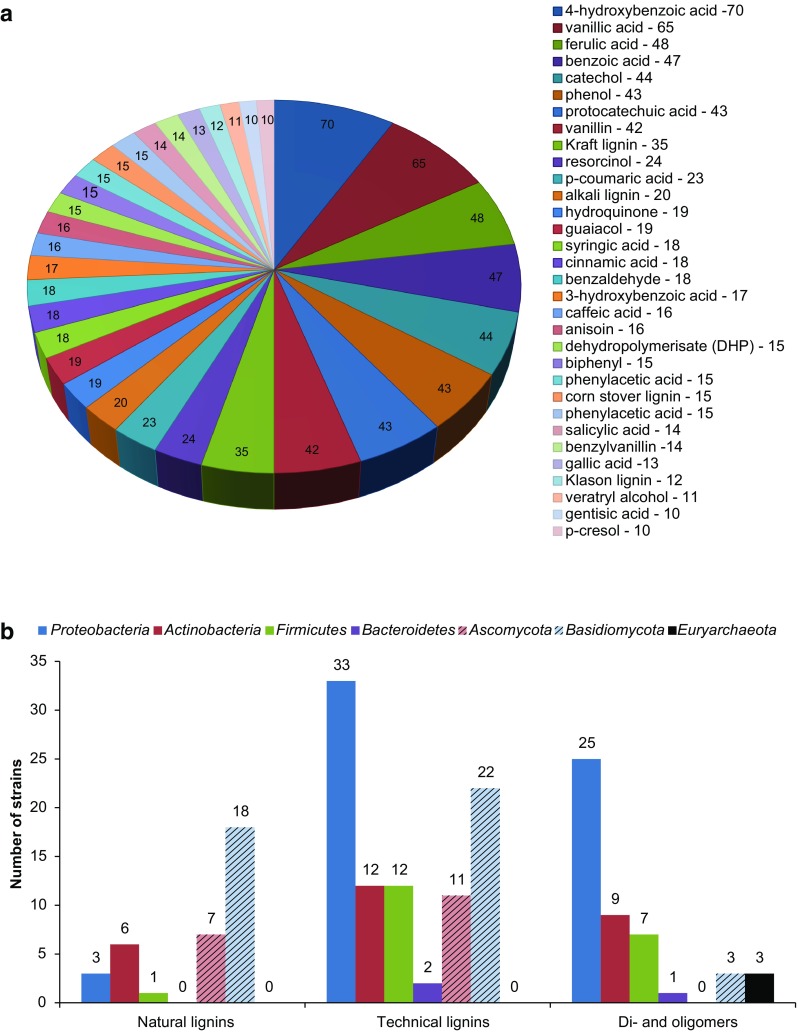
**a** Substrates that can be utilized by > 10 organisms listed in the database; the numbers represent the number of strains in the database that utilize each compound. Total number of substrates that satisfied the > 10 cutoff—32; total number of substrates in dataset—141. **b** Number of species that can degrade natural and technical lignins, and di- and oligomeric aromatic compounds, sorted by phylum. To distinguish the bacteria from the representatives of the other two kingdoms, the fungal phyla are presented with stripes and the only archaeal phylum is in solid black

Similar to how fundamental and applied studies on lignin focus on a few model organisms, many studies use a few common model aromatic model compounds that represent different funneling pathways (e.g., 4-hydroxybenzoic acid, vanillic acid, ferulic acid, *p*-coumaric acid, and benzoic acid) to evaluate the physiology of the microbial niche (see, e.g., Fischer et al. 2008; González et al. 1997; Kosa and Ragauskas 2012; Ravi et al. 2017; Vardon et al. 2015). However, from browsing eLignin, there appears to be a much higher substrate diversity in this niche than just these model compounds. This is illustrated in Fig. 4a, showing a meta-analysis of the “most popular” substrates in the eLignin bibliome in terms of the number of different microbes that have been reported in the literature to degrade them. Evidently, the model aromatics are in the top, which both suggest that they indeed are good model compounds for the different funneling pathways and that they have been popular choices for the experimental work that has been published on this topic. In addition, some natural and technical lignins (corn stover, kraft, Klason, and alkaline lignin), “synthetic” oligoaromatics (dehydropolymerisate), and dimers (biphenol, benzylvanillin) are among these top 32 substrates (Fig. 4a). The number of microbes in the database that have been reported to degrade natural and technical lignins and di-/oligoaromatics is presented in Fig. 4b. The results show that fungi are the most prevalent degraders of natural lignins, which is reasonable given the high diversity of lignolytic fungi. The reported technical lignins include chemically modified lignin polymers as well as chemically depolymerized lignin (i.e., a mixture of both high (polymeric) and low molecular weight lignins (mono- and oligomers)) which explains the high number of bacteria that have been reported to grow on technical lignins. Di- and oligoaromatic compounds were primarily reported in *Proteobacteria* in eLignin, but this is likely a literature bias since (model) monoaromatic compounds tend to be more commonly studied across all phyla. Note that there are no *Acidobacteria* or *Spirocheates* in the eLignin bibliome that have been reported to degrade natural/technical lignins and di-/oligoaromatics.

It is equally important to know the substrates that cannot be used by a given organism, as this will give the limitations of its metabolism. In fact, many isolation papers both list substrates that can and that cannot support growth (for a few examples, see Bache and Pfennig 1981; Defnoun et al. 2000; Harwood and Gibson 1988; Song 2009) and thereby present a valuable hint to which funneling pathways can and cannot be expected in the organism. At the moment, the indexing in eLignin has been focused on the substrates that *can* be used, but a logical next step for the database development is to also include substrates that an organism cannot use.

### Prediction of funneling pathway distributions

Lignin consists of three primary building blocks known as monolignols that plants produce from the amino acid phenylalanine: sinapyl alcohol (called syringyl, or S, unit when incorporated in the lignin polymer), coniferyl alcohol (guaiacyl unit; G), and *p-*coumaryl alcohol (*p*-hydroxyphenyl unit; H) (Vanholme et al. 2010). The ratio of units in the polymer differs depending on the lignin source, with softwood consisting of mainly G units with a small fraction of H units, hardwood having a combination of almost exclusively S and G, and monocots all three (Gellerstedt and Henriksson 2008; Gosselink et al. 2010). Recent reports have also shown that a caffeyl alcohol homopolymer (caffeyl unit; C) can be found in seed coats of, e.g., vanilla orchard and some cacti species (Barsberg et al. 2018; Chen et al. 2012a). Consequently, the composition of aromatics in the depolymerized lignin will differ greatly between different lignocellulose feedstocks.

Following the S, G, and H types, three main funneling pathways for monoaromatic catabolism have been defined, based on which of the main lignin units (or derivatives thereof) they catabolize: the sinapyl branch (two methoxy groups), coniferyl branch (one methoxy group), and the *p-*coumaryl branch (no methoxy groups) (see Fig. 3). Within eLignin, these branches were further divided into one or more sequential pathways in order to better specify which reactions a species have been characterized with, i.e., a bacteria with a vanillin degradation pathway will not necessarily have the pathway for ferulic acid, although these pathways are sequential in the coniferyl branch. Microbial aromatic catabolism is also not limited to the S, G, and H funneling branches, meaning that there is a need for naming of other routes as well, including aromatics that are derived from other origins than lignins (e.g., other plant matter). Some examples include the caffeic acid, benzoyl, resorcinol, and cresol pathways (Fig. 3). Funneling pathways for di- and oligomeric aromatics, the study of which has started emerging in certain species (Bugg et al. 2011b; Kamimura et al. 2017), is another example of essential catabolic routes.

**Fig. 5:**
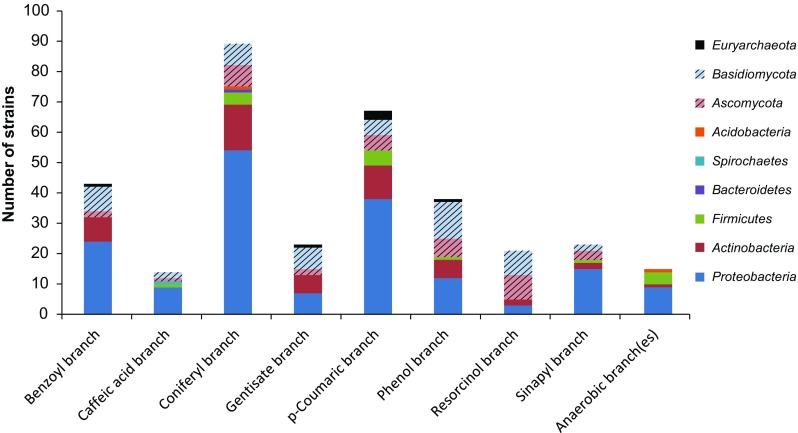
Distribution of putative funneling pathway branches in the eLignin bibliome, inferred from reported substrates, sorted by phylum. Because of the overall linear nature of the aerobic funneling pathways (Fig. 3), it is possible to use the substrates reported in the literature for a given organism and correlate that to a funneling pathway branch (i.e., a collection of funneling pathways). The small group of species that has been reported to degrade aromatics anaerobically has all been clustered in the anaerobic branch in order not to generate false positives in the other branches. To distinguish the bacteria from the representatives of the other two kingdoms, the fungal phyla are presented with stripes and the only archaeal phylum is in solid black. A detailed outcome of the prediction for each species with links to the different references is found online at www.elignindatabase.com under each organism entry page. Please note that the results are theoretical and it is up to everyone to assess the probability of these inferences, e.g., by reading the primary references for each organism

In a lot of bibliome studies, the substrate specificity of a species is presented without going into the intracellular conversion mechanisms nor reporting evidence of a specific funneling pathway. Therefore, in order to be able to use the eLignin dataset to look at pathway diversity, we developed a prediction algorithm to infer funneling branches from reported substrates from the literature. This is possible since many of the funneling branches are linear, e.g., ferulic acid is degraded via vanillin, and any species that have been reported to grow on these compounds and their intermediates can then be theoretically inferred to have the coniferyl branch (Fig. 3). Cinnamic acid is reported to be catabolized by 18 organisms (Fig. 4a), but due to the alternate metabolic routes for its degradation—e.g., via benzoic acid, 3-phenylpropionic acid, or styrene (Chamkha et al. 2001; Defnoun et al. 2000; Monisha et al. 2018; Mäkelä et al. 2015)—it was omitted from the prediction model. Also, according to current knowledge, anaerobic aromatic catabolism frequently (but not exclusively) relies on pathways that converge on benzoyl-CoA, that is further subjected to ATP-dependent hydrolysis to open the aromatic ring (Durante-Rodríguez et al. 2018; Fuchs et al. 2011); but since the exact mechanisms are largely unknown for the species in the dataset, all anaerobic microbes have been put in an “anaerobic branch (es)” cluster (Fig. 5).

The result of the theoretical prediction is presented in Fig. 5. The main conclusion is that, of the three main funneling branches (S, G, H), the coniferyl (G) and *p-*coumaric (H) branches seem by far to be the most abundant in niche 2. This might be correlated to the number of methoxy groups (none in the H unit, one in the G unit, two in the S unit; Fig. 3), as ring fission usually seems to occur after the methoxy groups have been demethylated to hydroxyl groups (Gupta et al. 1986; Nishikawa et al. 1998; Sampaio 1999). As the demethylation often requires a cofactor such as tetrahydrofolic acid (Masai et al. 2004) and NADH and FAD (Mallinson et al. 2018), the degradation of methylated aromatics may be limited by the rate of cofactor recycling. Furthermore, it is noteworthy that there is no caffeic acid degrading *Actinobacteria* yet in the eLignin database, despite the fact that they are the prokaryotic phylum that is commonly the second most abundant for most branches in the dataset (Fig. 5). Another observation is that metabolites of the resorcinol branch (Fig. 3) seem to be degraded by fungi to a larger extent than the other branches according to the current data (Fig. 5). Resorcinols are part of the phenolics in plants and soil humic acids (Burges et al. 1964; Kluge et al. 1990) and do not seem to be derived from lignin per se, which would put this compound within niche subgroup 2.

### Predicting organisms that can catabolize a given depolymerization mix

Many lignin valorization studies apply chemical depolymerization since microbial enzymatic breakdown of lignin is a very slow process taking many weeks (Fackler et al. 2006; Hedges et al. 1988; Liers et al. 2011). Therefore, from an applied point-of-view, it would be of interest to run the prediction model “backwards” in order to identify which organism(s) would be likely to grow on the mixture of aromatic monomers resulting from chemical depolymerization. The outcome of the depolymerization is highly dependent on process conditions and lignin source (Sun et al. 2018), and predicting the monomeric yield is beyond the scope of this review. However, the distribution of H, G, and S units in a given lignin might be indicative of the possible monomeric composition in the depolymerisate. Using this assumption, depolymerized softwood lignin would need microbes with funneling pathways for coniferyl- (G) and *p-*coumaric (H)-derived monomers. Spruce lignosulfonate has for instance been reported to yield vanillin, guaiacol, acetovanillone, and vanillic acid (Pérez and Tuck 2018). Some examples of organisms that can catabolize both vanillic acid and guaiacol include *Amycolatopsis* sp. ATCC 39116 (Pometto III et al. 1981), *Comamonas* sp. B-9 (Chen et al. 2012c), and *Rhodotorula rubra* IFO 889 (Huang et al. 1993). Hardwood depolymerisates would require species that can handle monomers derived from S and G units, and therefore, organisms with the syringyl (S) and coniferyl (G) branches would be needed, such as *Sphingobium* sp. SYK-6 (Katayama et al. 1988), *Acetobacterium woodii* NZva16 (Bache and Pfennig 1981), or *Rhizobium* sp. YS-1r (Jackson et al. 2017). Species that seem able to degrade compounds from all the S, G, and H branches, which would be representative of grass lignins, would include *Oceanimonas doudoroffii* JCM21046T (Numata and Morisaki 2015) and *Exophiala jeanselmei* CBS 658.76 (Middelhoven 1993). Please note that these predictions do not take culture and process conditions into account, meaning that some of these species might be better suited for process applications than others.

### Transport proteins

Although the chemical structure of many aromatic compounds allow them to passively diffuse through the lipid bilayers of biological membranes (Engelke et al. 1996), many microorganisms have dedicated transport channels or proteins for aromatic compounds—reviewed, e.g., by Parales and by Kamimura and their colleagues (Kamimura et al. 2017; Parales and Ditty 2017; Parales et al. 2008). In fact, transporter genes are commonly found within the catabolic operons for aromatic acids (Parales et al. 2008) which could suggest that the natural diffusion rate of certain aromatics is too limited for growth on aromatics as a sole carbon source. Transporters are of interest for metabolic engineering purposes, as a part of uptake optimization and/or expansion of the substrate range of a given strain. As more and more of the metabolic pathways for aromatic degradation are now elucidated, there seems to be an emerging effort within the fundamental molecular biology studies on lignin degradation to look into transport proteins. We have begun indexing transport proteins as part of the organism pages in eLignin, and we anticipate that this section will grow as this field expands.

Current knowledge on bacterial aromatic transporters mostly focuses on Gram-negative bacteria which have a cell envelope with two lipid bilayers separated by a periplasmic space: the outer and the inner membrane (Nikaido 2003). Some Gram-negatives have been reported to have substrate-specific diffusion channels for aromatic compounds on the outer membrane (Hearn et al. 2008; Nikaido 2003). Inner membrane transport of aromatic acids seems to be achieved by active transporters and not by diffusion in many species. This may be explained by the fact that these compounds are commonly protonated at neutral pH and—due to the hydrophobic charge—can partition into the membrane and damage the structure (Kamimura et al. 2017; Parales and Ditty 2017). Gram-negative bacteria with reported aromatic transporters include *Acinetobacter baylyi* ADP1 (Collier et al. 1997; D’Argenio et al. 1999), *Bradyrhizobium japonicum* USDA110 (Michalska et al. 2012), *Klebsiella pneumoniae* M5a1 (Xu et al. 2012), *Pseudomonas putida* KT2440 (Nishikawa et al. 2008) and PRS2000 (Nichols and Harwood 1997), *Rhodopseudomonas palustris* CGA009 (Giuliani et al. 2011; Michalska et al. 2012), *Sinorhizobium meliloti* 1024 (Michalska et al. 2012), and *Sphingobium* sp. SYK-6 (Mori et al. 2018). Gram-positives, on the other hand, only have a single cell membrane in their envelope: the cytoplasmic membrane (Parales and Ditty 2017). There seem to be less studies on Gram-positive than Gram-negative species with regard to aromatic transport. Some examples include *Corynebacterium glutamicum* ATCC 13032 (Chaudhry et al. 2007; Xu et al. 2006), *Lactobacillus plantarum* WCFS1 (Reverón et al. 2017), and *Rhodococcus jostii* RHA1 (Otani et al. 2014). It is also worthwhile to note that in addition to the mechanisms for transport of aromatics into the cell, many species also have efflux pumps in order to cope with the often cytotoxic properties of aromatic compounds (Parales and Ditty 2017), or as a means to excrete detoxified compounds. Ravi and colleagues have for instance described a *Pseudomonas* isolate that excreted vanillyl alcohol during growth on vanillin as a tolerance mechanism to handle excess vanillin that was not catabolized to vanillic acid fast enough, but the mechanism by which vanillyl alcohol was transported out of the cell has not been elucidated yet (Ravi et al. 2018).

## Conclusions and outlook

The interest for lignin as an underexploited carbon source has markedly increased during the last two decades, as evidenced by the exponential increase in published papers on lignin valorization (Abejón et al. 2018). In this minireview, we used our recently created resource, the eLignin database, to analyze the diversity of the lignin microbial niche, which we have defined as all microbes that can either degrade lignin or lignin-derived aromatic compounds. It should, however, be kept in mind that the data in eLignin encompasses the diversity in the bibliome, meaning that it reflects what people have reported in the literature. The papers that are indexed in the database concern microbial isolates, i.e., species that were cultivable. It is, therefore, inevitable that this approach does not represent the overall diversity of the lignin microbial niche, as there are many species within the niche community that cannot be detected and sustained with the common isolation methodologies. Although the aim of this minireview is to show the diversity of the niche, it also reveals the diversity and fashions within the scientific community, which may or may not correlate with the biological diversity. We can also conclude that the literature is enriched with physiological characterization, i.e., aromatic substrate specificities of different organisms are rather well known. The molecular biology of specific metabolic routes is, in contrast, less well elucidated, which will be an important next step both for the fundamental understanding of the biology and for the many projects that apply microbes in a value chain for lignin valorization. The prediction algorithm for aromatic pathways presented in this review can hopefully generate new hypotheses on the molecular biology of the niche and pave the way for future studies.

The microbiological aspects of lignin and aromatics degradation have a long history with a vast bibliome, and the need for resources such as the eLignin database will continue to grow as the field expands. In the future, we expect to further implement in eLignin a number of discussed features including improved prediction algorithms, lignolytic communities, and substrates that cannot be converted by a given organism. Economically feasible lignin valorization will require advanced metabolic engineering and thorough knowledge on microbial physiology. In that context, the objective of eLignin is not only to generate new overviews of the field but also to fuel new research ideas and engineering strategies and thus become an operational tool for studies on the microbiological aspect of lignin degradation, catabolism, and valorization.
